# A Hybrid Positioning Strategy for Vehicles in a Tunnel Based on RFID and In-Vehicle Sensors

**DOI:** 10.3390/s141223095

**Published:** 2014-12-05

**Authors:** Xiang Song, Xu Li, Wencheng Tang, Weigong Zhang, Bin Li

**Affiliations:** 1 School of Instrument Science and Engineering, Southeast University, Nanjing 210096, China; E-Mails: sx2190105@163.com (X.S.); zhangwg@seu.edu.cn (W.Z.); 2 School of Mechanical Engineering, Southeast University, Nanjing 210096, China; E-Mail: tangwc@seu.edu.cn; 3 Key Laboratory of Technology on Intelligent Transportation Systems, Ministry of Transport, Beijing 100088, China; E-Mail: libin@itsc.com.cn

**Keywords:** vehicle positioning, sensor fusion, tunnel, RFID, interactive multiple model

## Abstract

Many intelligent transportation system applications require accurate, reliable, and continuous vehicle positioning. How to achieve such positioning performance in extended GPS-denied environments such as tunnels is the main challenge for land vehicles. This paper proposes a hybrid multi-sensor fusion strategy for vehicle positioning in tunnels. First, the preliminary positioning algorithm is developed. The Radio Frequency Identification (RFID) technology is introduced to achieve preliminary positioning in the tunnel. The received signal strength (RSS) is used as an indicator to calculate the distances between the RFID tags and reader, and then a Least Mean Square (LMS) federated filter is designed to provide the preliminary position information for subsequent global fusion. Further, to improve the positioning performance in the tunnel, an interactive multiple model (IMM)-based global fusion algorithm is developed to fuse the data from preliminary positioning results and low-cost in-vehicle sensors, such as electronic compasses and wheel speed sensors. In the actual implementation of IMM, the strong tracking extended Kalman filter (STEKF) algorithm is designed to replace the conventional extended Kalman filter (EKF) to achieve model individual filtering. Finally, the proposed strategy is evaluated through experiments. The results validate the feasibility and effectiveness of the proposed strategy.

## 1. Introduction

In the last decade, there has been a significant amount of progress in intelligent transportation systems (ITSs). This is largely attributed to the recent developments in vehicle positioning technologies [1]. In various vehicle guidance- and safety-related applications such as traveler information, route guidance, automatic emergency calls, freight management, advanced driver assistance, or electronic fee collection [1,2], the importance of positioning system accuracy and reliability has increasingly been emphasized.

Global positioning systems (GPSs) are the most widely used technology in vehicle positioning nowadays [1,3,4]. However, a GPS is unable to provide accurate and reliable navigation solutions in the presence of signal interruption or blockage such as occurs in a tunnel [2,3,5,6]. With the rapid development of three-dimensional transportation, more and more tunnels have been increasingly constructed on highways or in cities. How to achieve accurate, reliable positioning in these tunnele has become a huge challenge for vehicle navigation [7].

To overcome the GPS signal blockage in a tunnel, a common solution is to integrate that GPS with an inertial navigation system (INS) due to the complementary nature of these two types of sensors [5,8,9]. However, only low-cost inertial sensors based on microelectromechanical system (MEMS) technology are affordable [10] for automotive applications due to the high cost of accurate INS systems. As an alternative, dead reckoning (DR) has been integrated with GPS for vehicle positioning [11]. However, INS and DR will accumulate large errors over time due to factors such as the measurement errors of MEMS-based inertial sensors and the characteristics of the integration process. These large errors can cause the rapid performance degradation during GPS outages [12,13], especially in the long tunnel. Other sensors, such as vehicle motion sensors [14,15], cameras [16] or radar, are used to compensate for the errors. These methods can partially correct the accumulated errors by INS or DR, but the compensation effect is poor when GPS is in a long-time failure scenario. The main reason is the lack of position observations to correct the errors.

Since GPS is usually not available for a long time in a tunnel, the GPS-based positioning technologies mentioned above always lead to incorrect positioning information, and are not suited to the tunnel environment. As an alternative, there has been rapid development of wireless location technologies [17–20] in recent years, such as Wireless Local Area Networks (WLAN), Bluetooth, Ultra Wide Band (UWB) and Radio Frequency Identification (RFID). Among them, RFID has attracted wide attention and research efforts due to its advantages of being non-contact, low-cost, high accuracy, with long-distance communication and the capability of working in a variety of harsh environments [21–23]. RFID is an automatic identification technology that relies on remotely storing and retrieving data using tags and readers. Although the original purpose of RFID technology was to identify objects [24], it has become a possible solution to obtain the object's location information in recent years, especially in the field of indoor positioning. RFID-based indoor positioning technology typically employs the received signal strength (RSS), the time of arrival (TOA), the time difference of arrival (TDOA) or angle of arrival (AOA) to compute an object's location [23,25,26].

With the rapid development of RFID-based technology, it has also been studied for outdoor applications. Chon applied RFID technology to the vehicle positioning field for the first time, and the feasibility of RFID-based vehicle positioning at high speed was preliminarily verified [27]. Zhang used the Active RFID Positioning (ARP) technology to achieve vehicle positioning [28]. Yang and Cheng employed passive RFID tags for vehicle navigation [29,30]. In these methods mentioned above, only the RFID information is used. Therefore, the positioning accuracy and the output frequency are not high enough to meet the performance requirements for many ITS applications [31]. In addition, these methods can only provide the position information, but they cannot provide the speed or attitude information which is also important for the location-related services.

To improve the performance of the RFID-based positioning method, the multi-sensor fusion method provides us a viable solution whereby the RFID information can be fused with that from several in-vehicle sensors. However, to the authors' knowledge, the fusion of the RFID technique and in-vehicle sensors has seldom been discussed in the related literature.

Several information fusion algorithms have been proposed in the field of vehicle positioning and navigation [12–16], such as extended Kalman filter (EKF) [1,2], unscented Kalman filter (UKF) [32], strong tracking Kalman filter (STKF) [6] and particle filter (PF) [33]. For these filtering methods, one important aspect that influences the positioning performance is the choice of an appropriate process model for the filter. In most of the literature, a single process model, whether simple or complicated, is built and utilized. However, in practice, it is very difficult to choose an optimal model to represent all driving situations. As an alternative, multiple model (MM) approaches [14,34] were proposed. These approaches assume that the system follows one of a finite number of different models. The possible vehicle driving patterns are represented by a set of models, and vehicle state information is obtained by combining specific model filters. Among several MM estimate methods, the interacting MM (IMM) [14] estimator is the most popular due to its high performance and low computational power requirements. Therefore, the IMM filter has been used for localization and tracking problems in several studies. In the actual implementation of IMM, EKF is the most widely used algorithm. However, it is well known that in conventional EKF, the system model, the system initial conditions, and the noise characteristics all have to be specified *a priori*. Therefore, in various circumstances, there are uncertainties in the noise description and system models due to the wide driving-maneuver range of vehicle operation, and the assumptions on the statistics of disturbances are violated since the availability of precisely known models is unrealistic in practical situations.

This paper aims to propose a multi-sensor fusion strategy based on RFID and in-vehicle sensors for vehicle positioning in a tunnel. In this strategy, the algorithms for both RFID-based preliminary positioning and global fusion are developed to obtain higher performance. The novel aspects of this paper can be summarized as follows:

(1) A RFID-based preliminary positioning algorithm is developed to provide preliminary position information for subsequent global fusion. In this algorithm, a Least Mean Square (LMS) [35] federated filter is designed to preliminary estimate the position of the vehicle. Compared to other RFID positioning algorithms, the proposed algorithm has many advantages, such as good fault-tolerance, and high precision, which can significantly improve the performance of the subsequent global fusion.
(2) An IMM-strong tracking extended Kalman filter (STEKF) algorithm is proposed to realize the global fusion. To overcome the disadvantages of the preliminary positioning algorithm, *i.e.*, low accuracy, low positioning frequency and the lack of speed or attitude information, low cost in-vehicle sensors, such as electronic compasses and wheel speed sensors, are introduced to fuse with the preliminary positioning results to extend the state, correct the preliminary positioning errors and improve the output frequency. Rather than single model-based filtering methods, the IMM-based algorithm considers the variety of driving conditions in which a vehicle can be operated and thus provides a better positioning accuracy. Meanwhile, to overcome the defects of the conventional EKF, the STEKF is developed to replace the EKF in the actual implementation of IMM to achieve model individual filtering.

The remainder of the paper is organized as follows: Section 2 gives an overview of the structure of the proposed multi-sensor fusion strategy. The preliminary positioning algorithm based on RFID is presented in Section 3. Section 4 presents the IMM-STEKF-based algorithm for further enhancing the positioning performance in the tunnel. Experimental results are provided in Sections 5. Section 6 is devoted to the conclusions.

## 2. Proposed Multi-Sensor Fusion Strategy

**Figure 1.**
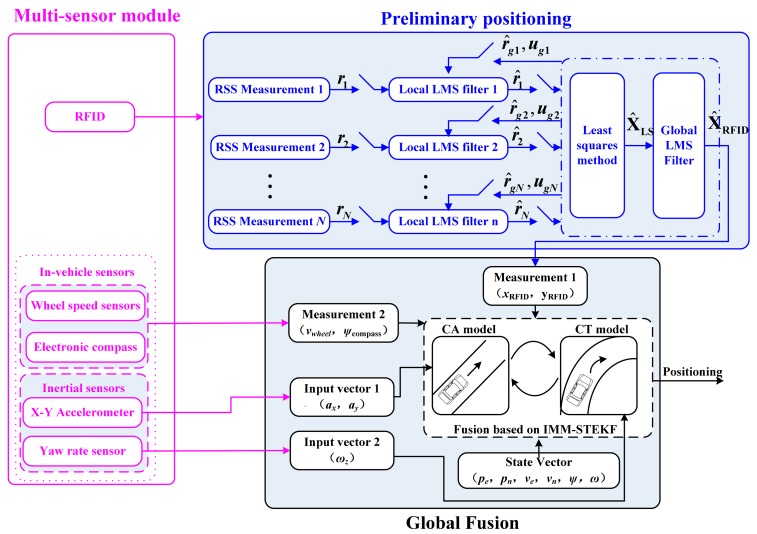
Proposed multi-sensor fusion strategy for vehicle positioning in a tunnel.

The proposed multi-sensor fusion strategy is mainly composed of three parts, *i.e.*, multi-sensor module, the preliminary positioning algorithm based on RFID, and the IMM-STEKF-based global fusion algorithm, as shown in Figure 1.

The multi-sensor module includes such sensors as MEMS-based inertial sensors, wheel speed sensors, electronic compass, and the low-cost active RFID hardware devices (a reader and a number of tags). The low-cost active RFID hardware devices are characterized by low price and low output frequency, typically 1 Hz. The MEMS-based inertial sensors used here only include two orthogonal accelerometers (*i.e.*, along the longitudinal and lateral axes in vehicle body frame) and a yaw gyro, rather than a full expensive INS. The wheel speed sensors and electronic compass are also low-cost sensors.

Because of the similarity between the tunnel and the indoor environment, the RFID technology can be used to achieve preliminary positioning for vehicles in the tunnel. The RSS-based location algorithms are the most widely used for indoor positioning because the algorithms are simple and need no additional hardware. This paper employs a two-step approach, namely, the calculation of the distances between the RFID tags and the reader based on RSS, and then the estimation of vehicle position. A decentralized LMS filter [35] in federated configuration is designed to estimate the position of the vehicle. Rather than other RFID positioning algorithms, the proposed LMS federated filter can effective improve the positioning accuracy and reliability in real world applications.

The IMM-STEKF algorithm is proposed to realize the fusion of the data from the preliminary positioning results and the low-cost in-vehicle sensors. The output frequency of fusion positioning is increased to 10 Hz from the 1 Hz value of the preliminary positioning. Meanwhile, the fusion algorithm can provide the speed and attitude information which RFID was unable to provide. The constant acceleration (CA) and constant turn (CT) models are adopted to represent two typical vehicle movements, *i.e.*, straight line and curvilinear motions, respectively. In the actual implementation of specific filtering of IMM, the STEKF algorithm is developed to replace the conventional EKF. For the vehicle, compared to the conventional EKF, it has the following advantages: (1) strong robustness toward changes of the actual system parameters during the vehicle operation process; (2) strong tracking ability during the mutation driving status; (3) lower sensitivity to system noise, measurement noise and the initial statistical properties [6,36]. By combining estimates from individual model-based STEKFs using the interacting process of the IMM filter, the fusion algorithm improves the accuracy and output frequency of positional information over a wide range of driving conditions in tunnels.

## 3. Preliminary Positioning Algorithm Based on RFID in the Tunnel

### 3.1. System Setup

**Figure 2.**
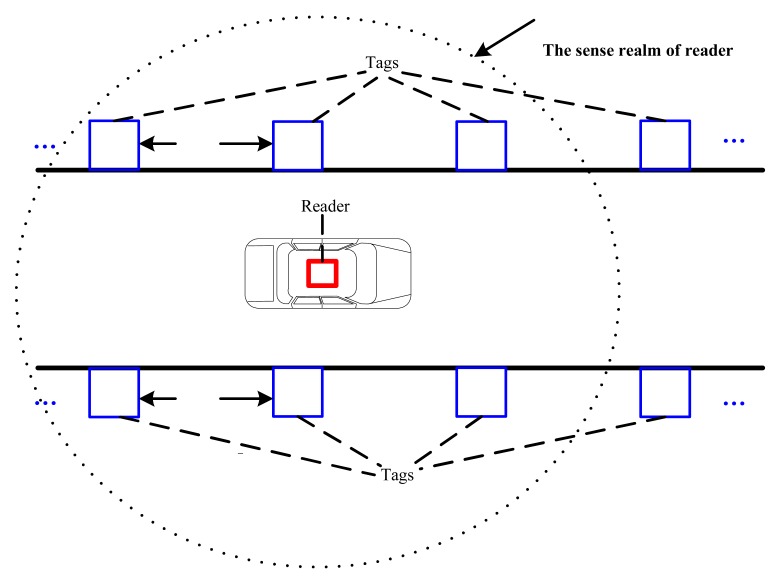
The RFID tag layout style.

An active RFID reader is installed on the top of the vehicle, and active RFID tags are placed on the both side walls of the tunnel, as shown in Figure 2. On each side, the tags are placed at regular intervals which is set according to the characteristics of the reader and tags to ensure at least four tags can be detected by reader at any moment. The exact position of each tag can be determined beforehand, and the position of the reader can be considered as the position of the vehicle.

### 3.2. The Distance Estimation Based on RSS

It seems that the first challenge of the two-step algorithm for preliminary positioning is how to mathematically model the relationship between the RSS and the distance. Theoretically, a propagation model can be applied to calculate the distance between the RFID reader and a tag according to the signal strength of the RFID tags. In unobstructed free space, the Friis transmission equation [23] shows that the signal strength level decreases at a rate inversely proportional to the distance traveled:

(1) $$P_{r}=P_{t}\frac{G_{t}G_{r}\lambda^{2}}{16L\pi^{2}r^{2}}$$

where *P_r_* is the power received by receiver antenna, *P_t_* is the power input to transmitter antenna, *G_t_* is transmitter antenna gain, *G_r_* is receiver antenna gain, *L* is system loss factor, *λ* is wavelength, and *r* is the distance between transmitter antenna and receiver antenna. Based on this relationship, we can estimate the distance between the RFID tag and the reader if the RSS is known. In real world applications, the parameters of this model must be determined in a specific environment by statistical analysis of the experimental data.

## 3.3. The Preliminary Positioning Algorithm Based on RFID

The second step is how to estimate the vehicle position based on the distances between reader and tags. For indoor location, the most widely used method is so-called multilateration method [23,25–27]. However, the values of calculation distance usually have a great error due to the failure of tags, which may cause large positioning errors. To solve the problem discussed above, a LMS-federated filter is designed as shown in Figure 1. In the federated filter, each local filter is a LMS filter. Assuming that the RSSs of *N* tags can be measured by the reader at the time *k*, the distance between each RFID tag and the reader, *r*_1_,*r*_2_,…,*r_N_*, can be calculated by Equation (1). To improve the accuracy of estimation distance, *N* local LMS filters are designed to further estimate the distances, as *r̂*_1_,*r̂*_2_,…,*r̂_N_*. Then the least squares (LS) algorithm is employed to estimate the position of reader, **X̂**_LS_. **X̂**_LS_ is filtered by using the global LMS filter, and the final estimation of position, **X̂**_RFID_, is output at time *k*. By utilizing **X̂**_RFID_, the distances between reader and tags, *r̂_g_*_1_,*r̂_g_*_2_,…,*r̂_gN_*, and the information distribution coefficients, *û_g_*_1_,*û_g_*_2_,…,*û_gN_*, are recalculated to update each local LMS filter. The significant advantage of this algorithm is that any false information from tags can be effectively isolated because of the federated filter structure.

LMS filter is a transverse filter [37]. *m_i_* is the transverse dimension of the *i*-th LMS filter. In each discrete time *k*, $v_{i}^{j}\left(j=0,1,\ldots ,m_{i}-1\right)$ represents the input of the *i*-th LMS filter at the moment (*k-j*). **z***_i_*(*k*) indicates the output of the *i*-th LMS filter at the moment *k*. Let:

(2) $${\text{v}}_{i}(k)={\left[\begin{array}{llllll} v_{i}^{0} & v_{i}^{1} & \cdots & v_{i}^{j} & \cdots & v_{i}^{m_{i}-1} \end{array}\right]}^{\prime }$$

The transverse weighted vector of the *i*-th LMS filter,**w***_i_*(*k*), is defined as:

(3) $${\text{w}}_{i}(k)={\left[\begin{array}{cccc} w_{i}^{0} & w_{i}^{1} & \cdots & w_{i}^{m_{i}-1} \end{array}\right]}^{\prime }$$

The calculations in the LMS filter algorithm at each step *k* are as follows:

(4) $${\text{z}}_{i}(k)={\text{w}}_{i}{\left(k\right)}^{\prime }{\text{v}}_{i}(k)$$

(5) $${\text{e}}_{i}(k)={\text{d}}_{i}(k)-{\text{z}}_{i}(k)$$

(6) $${\text{w}}_{i}(k+1)={\text{w}}_{i}(k)+{\text{u}}_{i}(k){\text{v}}_{i}(k){\text{e}}_{i}(k)$$

Assuming that (*x_i_, y_i_*) are the coordinates of the *i*-th tag, the coordinates of the reader estimated by LS algorithm is:

(7) $${\hat{\text{X}}}_{\text{LS}}(k)={\left[\begin{array}{cc} {\hat{x}}_{\text{LS}}(k) & {\hat{y}}_{\text{LS}}(k) \end{array}\right]}^{\prime }$$

And the finally estimation result of coordinate vector by global LMS filter is:

(8) $${\hat{\text{X}}}_{\text{RFID}}(k)={\left[\begin{array}{cc} {\hat{\text{X}}}_{\text{RFID}}(k) & {\hat{y}}_{\text{RFID}}(k) \end{array}\right]}^{\prime }$$

For each LMS filter, the values of variables are different in Equations (4)–(6). Let *i* = 0, 1, 2…, *N, i* = 0 represents the global filter, and *i* = 1, 2,…, *N* represent the local filters, respectively.

When *i* = 1, 2,…, N:

(9) $${\text{v}}_{i}(k)={\left[\begin{array}{llllll} r_{i}^{0} & r_{i}^{1} & \cdots & r_{i}^{j} & \cdots & r_{i}^{m_{i}-1} \end{array}\right]}^{\prime }$$

(10) $${\hat{r}}_{i}(k)={\text{z}}_{i}(k)$$

(11) $${\text{d}}_{i}(k)={\hat{r}}_{gi}(k)$$

(12) $${\text{u}}_{i}(k)=u_{gi}(k)$$

(13) $${\hat{r}}_{gi}=\sqrt{{\left({\hat{x}}_{\text{RFID}}\left(n\right)-x_{i}\right)}^{2}+{\left({\hat{y}}_{\text{RFID}}\left(n\right)-y_{i}\right)}^{2}}$$

When *i* = 0,

(14) $${\text{v}}_{0}(k)={\left[\begin{array}{llllll} {{\hat{\text{X}}}_{\text{LS}}}^{0} & {{\hat{\text{X}}}_{\text{LS}}}^{1} & \ldots & {{\hat{\text{X}}}_{\text{LS}}}^{j} & \ldots & {{\hat{\text{X}}}_{\text{LS}}}^{m_{0}-1} \end{array}\right]}^{\prime }$$

(15) $${\hat{\text{X}}}_{\text{RFID}}(k)={\left({\text{z}}_{0}\left(k\right)\right)}^{\prime }$$

(16) $${\text{d}}_{0}\left(k\right)={\left({\hat{\text{X}}}_{\text{RFID}}\left(k-p\right)\right)}^{\prime }$$

(17) $${\text{u}}_{0}\left(k\right)\leq 1$$

where **X̂**_LS_*^j^* is the estimation result by the LS at the moment (*k-j*), **u**_0_(*k*) and *p* are the filter parameters, **w***_i_*(0) is the initial weight of the *i*-th filter and can be set as:

(18) $${\text{w}}_{i}\left(0\right)={\left[\begin{array}{cccc} 1 & 1 & \ldots & 1 \end{array}\right]}^{\prime }/m_{i}$$

**u***_gi_*(*n*) is information distribution coefficients at the moment *k*. [**u***_g_*_1_(*k*) … **u***_gN_*(*k*)] can be designed as normalized vector:

(19) $$\left[\begin{array}{ccc} \frac{\left|{\hat{r}}_{1}\left(k\right)-{\hat{r}}_{g1}\left(k\right)\right|}{{\hat{r}}_{g1}\left(k\right)} & \cdots & \frac{\left|{\hat{r}}_{N}\left(k\right)-{\hat{r}}_{gN}\left(k\right)\right|}{{\hat{r}}_{gN}\left(k\right)} \end{array}\right]$$

The LS algorithm at each step *k* can be described as follows:

(20) $${\hat{\text{X}}}_{\text{RFID}}\left(k\right)={\left(\text{S}\prime \text{S}\right)}^{-1}\text{S}\prime \left(\lambda -{\hat{r}}_{1}\rho \right)$$

where:

$$\begin{array}{ccc} \text{S}=\left[\begin{array}{ll} x_{2}-x_{1} & y_{2}-y_{1}\\ \cdots & \cdots \\ x_{N}-x_{1} & y_{N}-y_{1} \end{array}\right], & \rho =\left[\begin{array}{c} {\hat{r}}_{2}-{\hat{r}}_{1}\\ \cdots \\ {\hat{r}}_{N}-{\hat{r}}_{1} \end{array}\right], & \lambda =\frac{1}{2}\left[\begin{array}{c} x_{2}^{2}+y_{2}^{2}-x_{1}^{2}-y_{1}^{2}-{\left({\hat{r}}_{2}-{\hat{r}}_{1}\right)}^{2}\\ \cdots \\ x_{N}^{2}+y_{N}^{2}-x_{1}^{2}-y_{1}^{2}-{\left({\hat{r}}_{N}-{\hat{r}}_{1}\right)}^{2} \end{array}\right] \end{array}$$

## 4. IMM-STEKF-Based Algorithm for Global Fusion

Since the preliminary positioning algorithm utilizing only RFID has some deficiencies as discussed above, the in-vehicle sensors are introduced to fuse with the preliminary positioning results to further enhance the positioning performance in the tunnel. In practice, a single vehicle model can hardly represent all possible vehicle motions. Therefore, it is preferable to use multiple models to represent different vehicle motions. In the field of tracking or localization, there are many approaches to combine multiple models. Among them, the IMM approach has been proven to be effective and efficient. In this section, the IMM-based algorithm is proposed to realize the fusion of the results of RFID-based preliminary positioning and the in-vehicle sensors. In the actual implementation of IMM, the STEKF algorithm is developed to replace the conventional EKF and thus overcome the defects of the conventional EKF in present study, which can be used to solve the state estimation problem of a class of nonlinear systems with white noise [6].

### 4.1. Vehicle Model Set

For vehicles in a tunnel, typical motion patterns include straight line (acceleration or deceleration), curve, and lane change *etc*. Generally, these patterns can be represented by a combination of a straight-line model and a curvilinear model.

To achieve a balance between model accuracy and computational complexity, the constant acceleration (CA) and constant turn (CT) models [34] are adopted in this paper to describe the straight-line and curvilinear motions, respectively. Both vehicle models have the same states as follows:

(21) $$\text{X}(k)={\left[\begin{array}{llllll} p_{e}\left(k\right) & p_{n}\left(k\right) & v_{e}\left(k\right) & v_{n}\left(k\right) & \psi \left(k\right) & \omega \left(k\right) \end{array}\right]}^{\prime }$$

where *p_e_*(*k*) and *p_n_*(*k*) represent the east and north coordinates of the vehicle CoG in the global positioning frame (*i.e*., e-frame), respectively. *v_e_*(*k*) and *v_n_*(*k*) represent the east and north velocity. *Ψ*(*k*) and *ω*(*k*) represent the yaw angle and yaw rate. *k* is the discrete-time step. The CA model equation is shown as follows:

(22) $${\text{X}}_{1}\left(k,k-1\right)={\text{f}}_{1}\left({\text{X}}_{1}\left(k-1\right),{\text{U}}_{1}\left(k\right)\right)=\left[\begin{array}{c} p_{e}\left(k-1\right)+v_{e}\left(k-1\right)T+\frac{1}{2}\left[a_{x}\cos\psi \left(k-1\right)-a_{y}\sin\psi \left(k-1\right)\right]T^{2}\\ v_{e}\left(k-1\right)+\left[a_{x}\cos\psi \left(k-1\right)-a_{y}\sin\psi \left(k-1\right)\right]T\\ p_{n}\left(k-1\right)+v_{n}\left(k-1\right)T+\frac{1}{2}\left[a_{x}\sin\psi \left(k-1\right)+a_{y}\cos\psi \left(k-1\right)\right]T^{2}\\ v_{n}\left(k-1\right)+\left[a_{x}\sin\psi \left(k-1\right)+a_{y}\cos\psi \left(k-1\right)\right]T\\ \psi \left(k-1\right)\\ 0 \end{array}\right]$$

where **f**_1_(·) denotes the process function of the CA model. *T* is the sampling interval. **X**_1_(*k*,*k*−1) = **X**(*k*,*k*−1) and **U**_1_(*k*) = [*a_x_ a_y_*]^′^ are the state and input vector, respectively. *a_x_* and *a_y_* denote the longitudinal and lateral accelerations in the vehicle body frame, which can be measured by the accelerometers.

The CT model equation is shown as follows:

(23) $${\text{X}}_{2}\left(k,k-1\right)={\text{f}}_{2}\left({\text{X}}_{2}\left(k-1\right),{\text{u}}_{2}\left(k\right)\right)=\left[\begin{array}{c} p_{e}\left(k-1\right)+v_{e}\left(k-1\right)\frac{\sin\omega_{z}T}{\omega_{z}}+v_{n}\left(k-1\right)\frac{\cos\omega_{z}T-1}{\omega_{z}}\\ v_{e}\left(k-1\right)\cos\omega_{z}T-v_{n}\left(k-1\right)\sin\omega_{z}T\\ p_{n}\left(k-1\right)+v_{e}\left(k-1\right)\frac{1-\cos\omega_{z}T}{\omega_{z}}+v_{n}\left(k-1\right)\frac{\sin\omega_{z}T}{\omega_{z}}\\ v_{e}\left(k-1\right)\sin\omega_{z}T+v_{n}\left(k-1\right)\cos\omega_{z}T\\ \psi \left(k-1\right)+\omega_{z}T\\ \omega \left(k-1\right) \end{array}\right]$$

where **f**_2_(·) denotes the process function of the CT model. **X**_2_(*k*,*k*−1) = **X**(*k*,*k*−1) and **U**_2_(*k*) = [*ω_z_*] are the state and input vector, respectively. *ω_z_* is the yaw rate which can be measured by the yaw gyro.

### 4.2. Observation Model

As shown in Figure 1, the observation information comes from two sources, *i.e.*, RFID preliminary positioning results and the data from in-vehicle sensors. The observation equation can be established as

(24) $$\text{z}=\text{h}\left[\text{X}\left(k\right)\right]=\left[\begin{array}{c} p_{e}\left(k\right)+n_{pe}\\ p_{n}\left(k\right)+n_{pn}\\ v_{e}\left(k\right)\cos\psi \left(k\right)+v_{n}\left(k\right)\sin\psi \left(k\right)+n_{v}\\ \psi \left(k\right)+n_{\psi } \end{array}\right]$$

where **Z** = [*x*_RFID_ *y*_RFID_ *v*_RFID_ *Ψ*_compass_]^′^ is the observation vector and **h** is the corresponding observation function. *x*_RFID_ and *y*_RFID_ are the east and north positions observed by the RFID, *v_wheel_* is the longitudinal linear velocity in the vehicle body frame, which can be calculated by wheel speed sensors, *Ψ_compass_* is the observed yaw angle by the electronic compass. *n_pe_, n_pn_, n_v_* and *n_Ψ_* denote the corresponding observation noise vectors with the assumption of the zero-mean and Gaussian distribution.

### 4.3. Global Fusion Algorithm Based on IMM-STEKF

**Figure 3.**
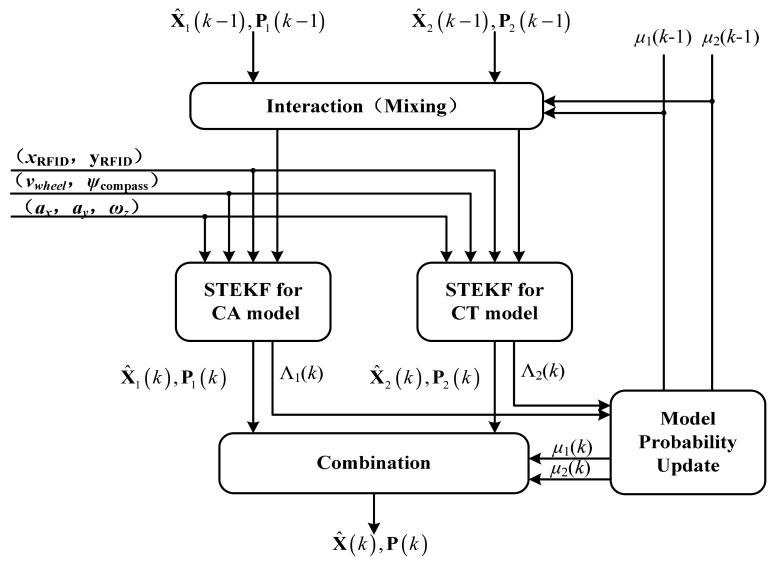
The IMM-STEKF algorithm.

Using the vehicle model set and observation model discussed above, the IMM-STEKF algorithm is used to achieve a precise estimation of the vehicle's position in the tunnel. The IMM algorithm mainly includes the following four steps, as shown in Figure 3.

(1) Interaction:
  The individual filter estimate **X̂***_i_*(*k*−1) of the *i*-th vehicle model (*i* = 1,2) is mixed with the other data using the predicted model probability *μ_i_*(*k*−1), and the Markov transition probability *π_ji_, i.e.*, the probability that the transition occurs from state *j* to state *i*:
  (25) $$\begin{array}{cc} \mu_{i}\left(k,k-1\right)=\sum_{j=1}^{2}\pi_{ji}\mu_{j}\left(k-1\right) & \left(i=1,2\right) \end{array}$$
  The mixing weight is given by:
  (26) $$\begin{array}{cc} \mu_{ji}\left(k-1\right)=\pi_{ji}\mu_{j}\left(k-1\right)/\mu_{i}\left(k,k-1\right) & \left(i,j=1,2\right) \end{array}$$
  The mixing of the state estimates **X̂***_j_*(*k*−1) and theirs covariances **P**(*k*−1) can be computed as:
  (27) $$\begin{array}{cc} {\bar{\text{X}}}_{i}\left(k-1\right)=\sum_{j=1}^{2}\mu_{ji}\left(k-1\right){\hat{\text{X}}}_{j}\left(k-1\right) & \left(i=1,2\right) \end{array}$$
  (28) $${\bar{\text{P}}}_{i}\left(k-1\right)=\sum_{j=1}^{2}\mu_{j|i}\left(k-1\right)\left\{{\text{P}}_{j}\left(k-1\right)+\left[{\bar{\text{X}}}_{i}\left(k-1\right)-{\hat{\text{X}}}_{j}\left(k-1\right)\right]\;{\left[{\bar{\text{X}}}_{i}\left(k-1\right)-{\hat{\text{X}}}_{j}\left(k-1\right)\right]}^{\prime }\right\}\left(i=1,2\right)$$
(2) Model individual filtering based on STEKF
  Each filter predicts and updates its state and covariance by using its corresponding model. Rather than EKF, the STEKF is adopted to execute this step in the present study because the performance of conventional EKF can be easily influenced by improper input data. Sub-optimal fading factors are introduced into the nonlinear smooth algorithm in the STEKF algorithm. For the model described by Equations (22)–(24), the specific algorithm of STEKF can be illustrated as follows:
  (29) $$\begin{array}{cc} {\hat{X}}_{i}\left(k,k-1\right)={\text{f}}_{i}\left({\overset{\mathrm{macr}}{X}}_{i}\left(k-1\right),{\text{U}}_{i}\left(k\right)\right) & \left(i=1,2\right) \end{array}$$
  (30) $$\begin{array}{cc} {\text{g}}_{i}\left(k\right)=\text{Z}\left(k\right)-\text{h}\left({\hat{\text{X}}}_{i}\left(k,k-1\right)\right) & \left(i=1,2\right) \end{array}$$
  (31) $${\text{P}}_{i}\left(k,k-1\right)=\lambda_{i}\left(k\right){\text{A}}_{i}\left(k,k-1\right){\bar{\text{P}}}_{i}\left(k-1\right){\text{A}}_{i}^{\prime }\left(k,k-1\right)+{\text{B}}_{i}\left(k,k-1\right)\Gamma_{i}\left(k-1\right){\text{B}}_{i}^{\prime }\left(k,k-1\right)+{\text{Q}}_{i}\left(k\right)$$
  (32) $$\begin{array}{cc} {\text{K}}_{i}\left(k\right)={\text{P}}_{i}\left(k,k-1\right)\cdot {\text{H}}^{\prime }\left(k\right)\cdot {\left[\text{H}\left(k\right){\text{P}}_{i}\left(k,k-1\right){\text{H}}^{\prime }\left(k\right)+\text{R}\left(k\right)\right]}^{-1} & \left(i=1,2\right) \end{array}$$
  (33) $$\begin{array}{cc} {\hat{\text{X}}}_{i}\left(k\right)={\hat{\text{X}}}_{i}\left(k,k-1\right)+{\text{K}}_{i}\left(k\right){\text{g}}_{i}\left(k\right) & \left(i=1,2\right) \end{array}$$
  (34) $$\begin{array}{cc} {\text{P}}_{i}\left(k\right)=\left[\text{I}-{\text{K}}_{i}\left(k\right)\cdot \text{H}\left(k\right)\right]\cdot {\text{P}}_{i}\left(k,k-1\right) & \left(i=1,2\right) \end{array}$$
  where **A***_i_*(*k, k*−1) and **B***_i_*(*k, k*−1) are the Jacobian matrices of the process function **f***_i_*(·) with respect to **X̅***_i_* and **U***_i_*, **P***_i_*(*k, k*−1) is the covariance of the state prediction, **Γ***_i_*(*k*−1) and **Q***_i_*(*k*) are the covariance matrices of the process noise and the input noise, respectively. **K***_i_*(*k*) is the Kalman gain associated with the observation sensors, **H**(*k*) is the Jacobian matrice of the observation function, **R**(*k*) is the covariance matrix of the measurement noises, **P***_i_*(*k*) is the estimation error covariance. *λ_i_*(*k*) = *diag*[*λ_i_*_1_(*k*), *λ_i_*_2_(2*k*),…, *λ_im_*(*k*)] is time-varying fading matrix; *λ_ij_*(*k*) (*j* = 1,2,…,*m*) are time varying fading factors. Based on the orthogonal principle of new information sequence, how to determine the time varying fading factor can be summarized to an unconstrained multivariate non-linear programming problem. The approximate calculation method of *λ_ij_*(*k*) is shown as follows:
  (35) $$\lambda_{ij}\left(k\right)=\{\begin{array}{ll} \lambda_{0i}, & \lambda_{0i}\geq 1\\ 1, & \lambda_{0i}<1 \end{array}$$
  where:
  (36) $$\lambda_{0i}=tr\left[{\text{N}}_{i}\left(k\right)\right]/\left[{\text{M}}_{i}\left(k\right)\right]$$
  (37) $${\text{S}}_{i}\left(k\right)=\{\begin{array}{l} \begin{array}{ll} g_{i}\left(0\right)g_{i}^{\prime }\left(0\right) & k=0 \end{array}\\ \begin{array}{ll} \left[\rho {\text{S}}_{i}\left(k-1\right)+g_{i}\left(k\right)g_{i}^{\prime }\left(k\right)\right]/\left(1+\rho \right) & k\geq 1 \end{array} \end{array}$$
  (38) $${\text{N}}_{i}\left(k\right)={\text{S}}_{i}\left(k\right)-\text{H}\left(k-1\right){\text{Q}}_{i}\left(k-1\right){\text{H}}^{\prime }\left(k-1\right)-\beta \text{R}\left(k\right)$$
  (39) $${\text{M}}_{i}\left(k\right)=\text{H}\left(k-1\right){\text{A}}_{i}\left(k-1\right){\text{P}}_{i}\left(k-1\right){\text{A}}_{i}^{\prime }\left(k-1\right){\text{H}}^{\prime }\left(k-1\right)$$
  where 0 < *ρ* ≤ 1 stands for forgetting factor. *β* ≥ 1 stands for softening factor. In this paper, *ρ* and *β* are selected by experience. When the state mutated, the error variance matrixes **S***_i_*(*k*) increased due to the accretion of estimation error $g_{i}\left(k\right)g_{i}^{\prime }\left(k\right)$, then the corresponding time varying factor is increased and the tracking ability of filter will be enhanced.
(3) Model Probability Update
  Each model probability is updated according to the innovation error. Assuming Gaussian statistics, the likelihood for the observation can be calculated as follows:
  (40) $$\begin{array}{cc} \Lambda_{i}\left(k\right)=\frac{\exp\left\{-\frac{1}{2}{\left[\text{Z}\left(k\right)-\text{h}\left({\hat{\text{X}}}_{i}\left(k,k-1\right)\right)\right]}^{\prime }\left({\text{S}}_{i}{\left(k\right)}^{-1}\right)\left[\text{Z}\left(k\right)-\text{h}\left({\hat{\text{X}}}_{i}\left(k,k-1\right)\right)\right]\right\}}{\sqrt{\left|2\pi {\text{S}}_{i}\left(k\right)\right|}} & \left(i=1,2\right) \end{array}$$
  The model probability update is calculated as:
  (41) $$\begin{array}{cc} \mu_{i}\left(k\right)=\frac{\mu_{i}\left(k,k-1\right)\Lambda_{i}\left(k\right)}{\sum_{j=1}^{2}\mu_{j}\left(k,k-1\right)\Lambda_{j}\left(k\right)} & \left(i=1,2\right) \end{array}$$
  Note that in Equation (41), *μ*_1_ is the model probability of CA, while *μ*_2_ denotes the model probability of CT. in other words, *μ*_1_ and *μ*_2_ can represent the degree of dependence on the CA model and the CT model, respectively.
(4) Combination
  The combined state **X̂**(*k*) and its covariance matrix **P**(*k*) can be calculated as:
  (42) $$\hat{\text{X}}\left(k\right)=\sum_{i=1}^{2}\mu_{i}\left(k\right){\hat{\text{X}}}_{i}\left(k\right)$$
  (43) $$\text{P}\left(k\right)=\sum_{i=1}^{2}\mu_{i}\left(k\right)\left\{{\text{P}}_{i}\left(k\right)+\left[\hat{\text{X}}\left(k\right)-{\hat{\text{X}}}_{i}\left(k\right)\right]{\left[\hat{\text{X}}\left(k\right)-{\hat{\text{X}}}_{i}\left(k\right)\right]}^{\prime }\right\}$$

## 5. Experimental Results

**Figure 4.**
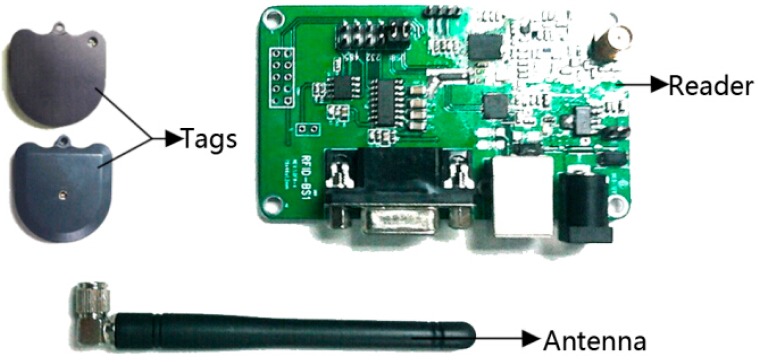
RFID hardware devices for the vehicle positioning experiment.

To verify the positioning performance of the proposed strategy, experiments were conducted on a Buick Sail SRV vehicle. It was equipped with Crossbow MEMS-based IMU-440 inertial sensors sampled at 100 Hz as well as wheel speed sensors sampled at 100 Hz and an electronic compass sampled at 10 Hz. The sensor accuracies (1σ) are 0.1 m/s^2^ for the accelerometers, and 0.2 °/s for the yaw rate sensor. Moreover, an accurate differential GPS (DGPS) NovAtel L1L2/RT2 was used as a reference for performance evaluation. In the experiment, the RFID hardware devices, as shown in Figure 4, included one NWR-01 RFID reader with an antenna, and a number of NWI-01 active RFID tags (417.05∼435.9 MHz, −8∼13 dBm).The RSS range of tag is normalized to 0–255, and the maximum measured distance of tag is 9 m.

**Figure 5.**
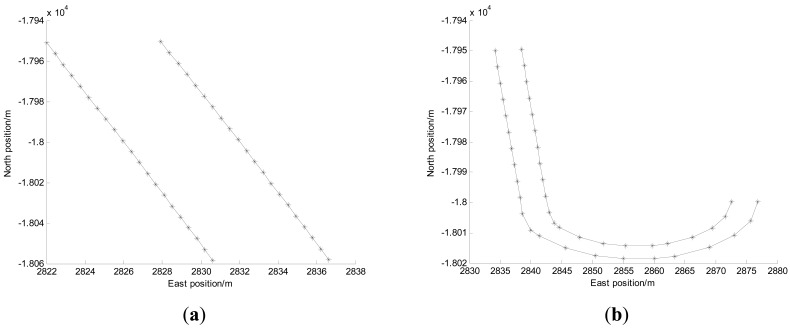
Setting the shapes of the tunnels: (**a**) Straight tunnel (**b**) Curved tunnel.

Because of experimental condition limitations, the simulated tunnels were set in an outdoor open space where the reference trajectory can be obtained by DGPS. The width of the tunnels are set to 7.5 m according to the width of two-lane tunnel, and the shapes of the tunnels are respectively set to the straight and curve in order to evaluate the performance of IMM algorithm, as shown in Figure 5.

In Figure 5, the “*” symbols represent the active RFID tags which are placed on both sides of the tunnel edge. The gap along the direction of the tunnel between adjacent tags is about 6 m. During the experiments, all sensor data were collected, and then the positioning algorithms were evaluated using the logged data.

### 5.1. Modeling of the Relationship between RSS and Distance

**Figure 6.**
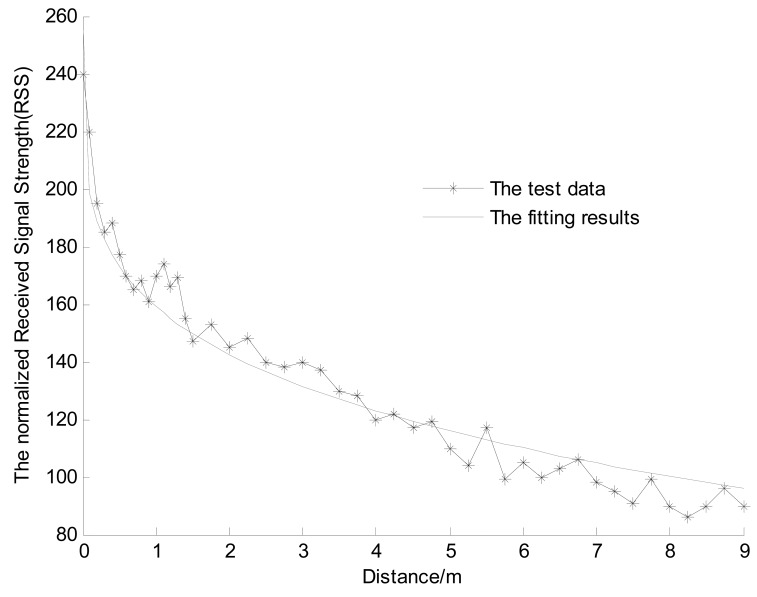
The fitting results of the relationship in outdoor test site.

In different situations (such as the laboratory, outdoor test site and tunnel), we fit the curve that shows the relationship of RSS with distance between the reader and the active tag using Equation (1). The RSSs are collected at different distances between the tag and the reader. In 0–1.5 m range, the collection gap is 0.1 m, and in the 1.5–9 m range, the collection gap is 0.25 m. There are four tags at each same collection location, and the collection time is 5 min. The average value of RSS is considered as the true RSS value of this location. The fitting results of the relationship between RSS and distance at the outdoor test site are shown in Figure 6. The mean and standard deviation (STD) of the RSS fitting errors in different situations are shown in Table 1.

### 5.2. Performance of RFID-Based Preliminary Positioning Algorithm

**Figure 7.**
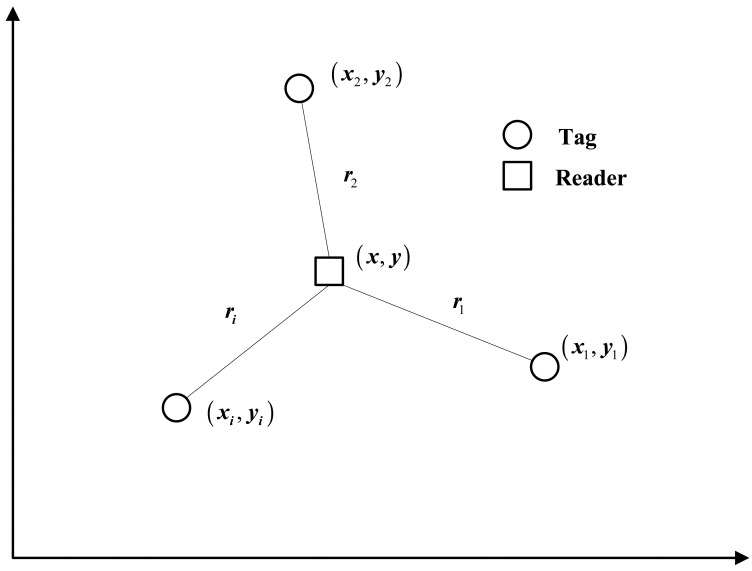
Schematic of the multilateration method.

To evaluate the effect of the preliminary positioning algorithm (abbreviated as LMS-Federated) discussed above, the multilateration method [23,25–27] is also investigated for comparison. The multilateration method is the most widely used method for indoor location. Figure 7 shows the schematic of 2-D localization using multilateration, and it can be easily extended to 3-D space.

From Figure 7, if there are *i* tags (*i* >= 3) with known coordinates (*x_i_, y_i_*), and the distances between the reader with unknown coordinates (*x, y*) and tags are estimated to be *r_i_*, we can obtain:

(44) $$\{\begin{array}{c} r_{1}^{2}={\left(x-x_{1}^{2}\right)}^{2}+{\left(y-y_{1}^{2}\right)}^{2}\\ r_{2}^{2}={\left(x-x_{2}^{2}\right)}^{2}+{\left(y-y_{2}^{2}\right)}^{2}\\ \cdots \\ r_{i}^{2}={\left(x-x_{i}^{2}\right)}^{2}+{\left(y-y_{i}^{2}\right)}^{2} \end{array}$$

**Figure 8.**
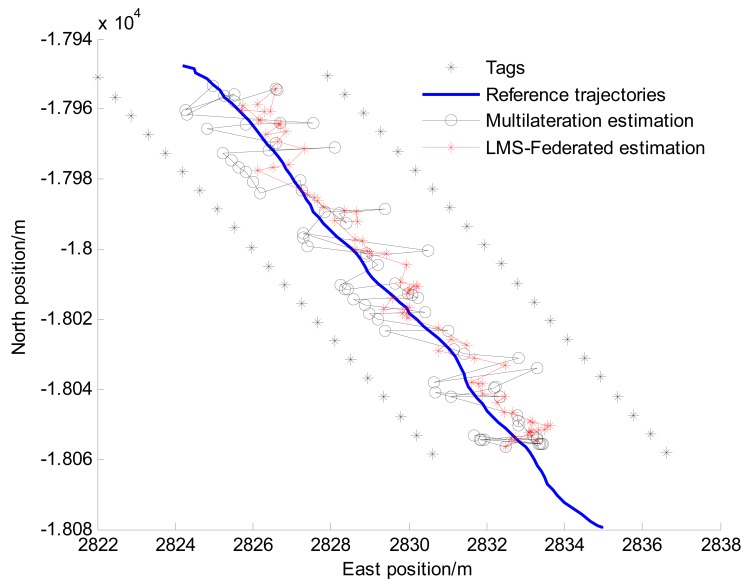
The vehicle trajectories.

**Figure 9.**
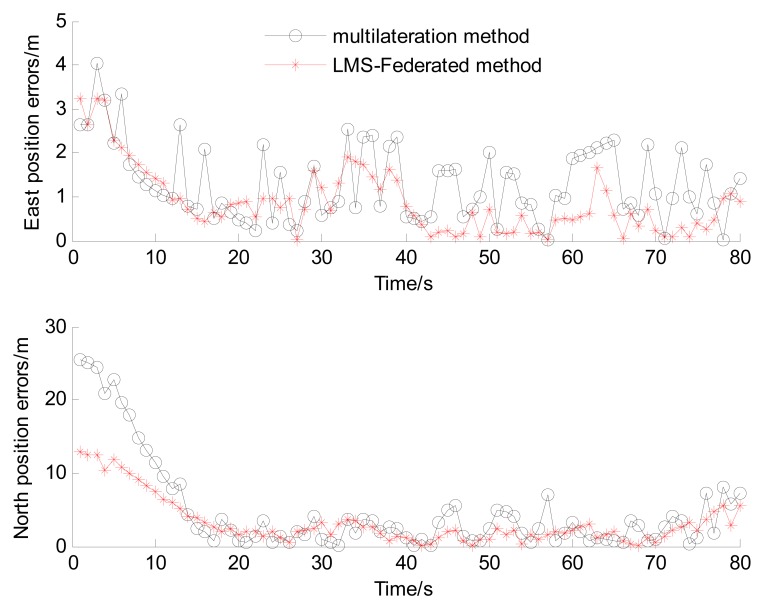
The east and north positioning errors.

By solving this equation group, the coordinates of the reader can be calculated. The reference and estimated vehicle trajectories in an experiment are shown in Figure 8, the east and north positioning errors are shown in Figure 9.

From Figures 8 and 9, it can be seen that the performance of LMS-Federated is obviously improved compared with that of multilateration. However, From Figure 9, we can find that the positioning errors are large when the vehicle is outside the tunnel (0–15 s), because the reader can't detect enough tags in these areas, *i.e.*, the number of tags is less than 3. Table 2 shows statistics of Euclidean distance errors when the vehicle is driving in the tunnel (15–80 s).

From Table 2, we can find that when the vehicle is driving in the tunnel, the RMS value of Euclidean distance error of LMS-Federated algorithm is decreased to 3.01 m, *i.e.*, about 47% accuracy improvement over the multilateration method. The reason is that the accuracy of the estimated distance between reader and tags can be improved by the local LMS filter, and the error or failed RSS information from tags can be effectively isolated.

This performance can meet the positioning requirement of actual driving situations since the GPS is available outside the tunnel. Further statistics and analysis of the preliminary positioning results will be described in the next section.

### 5.3. Evaluation of IMM-STEKF-Based Fusion Positioning Performance

#### 5.3.1. Straight Line Driving Test

**Figure 10.**
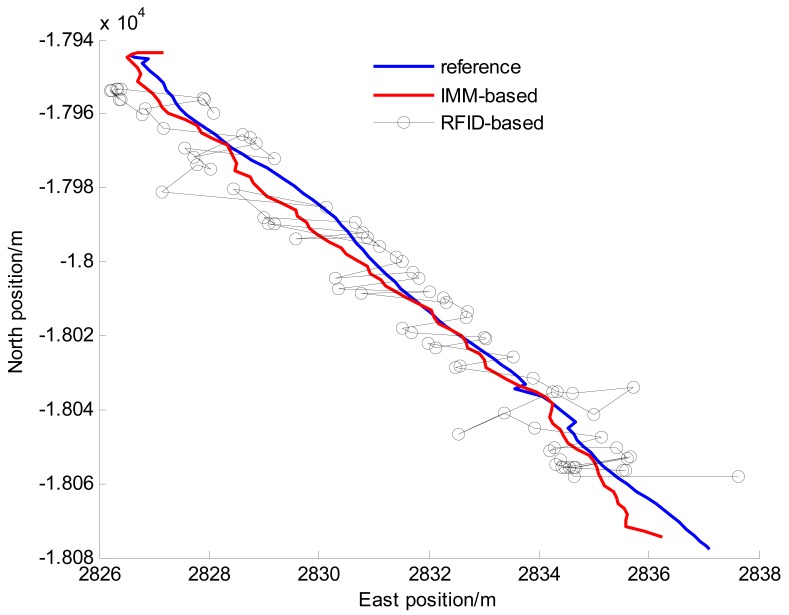
The vehicle trajectories.

**Figure 11.**
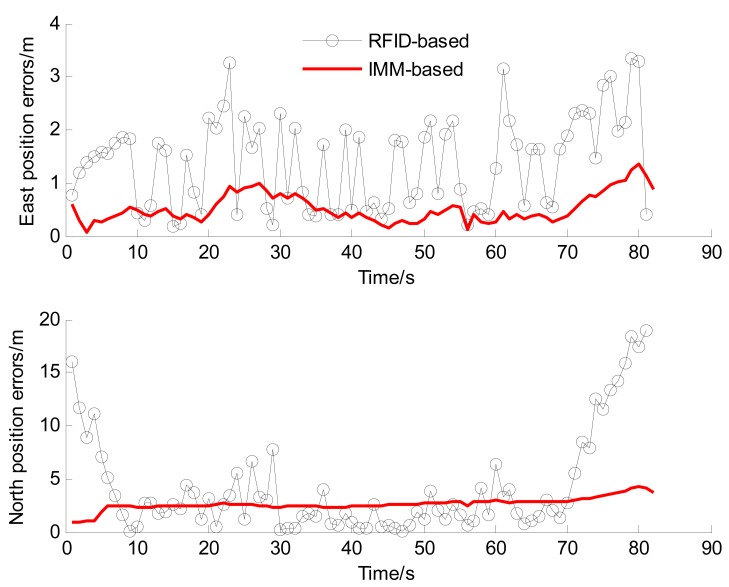
The east and north positioning errors.

For preliminary validation and evaluation of the performance of proposed fusion positioning algorithm, a total of nine straight line driving tests have been carried out, and the additional comprehensive tests are described in Section 5.2.2. The straight line driving test situations include acceleration, deceleration and uniform motion under different vehicle speed conditions. For brevity, only one test is shown here as an example because similar conclusions can be reached from the other tests. The time of the test is 81 s, and the frequency of RFID-based preliminary positioning is 1 Hz. The reference and estimated vehicle trajectories in the experiment are shown in Figure 10. Figure 11 gives the positioning errors of the proposed preliminary positioning based on LMS-federated filter (abbreviated as RFID-based) and the global fusion positioning (abbreviated as IMM-based).

From Figure 10, it can be seen the estimated position of vehicle is approximately the reference position. From Figure 11, we can find that the preliminary positioning errors are large when the vehicle is outside the tunnel (0–5 s and 75–81 s), and when the vehicle is driving in the tunnel (6–74 s), the positioning error is relatively small. The mean and standard deviation of the east and north positioning errors are shown in Table 3. Table 4 gives the Euclidean distance error statistics (*i.e.*, horizontal position errors). In order to illustrate the effectiveness of the fusion algorithm for positioning in the tunnel, the positioning performances at the stage when the vehicle driving in the tunnel completely (6–74 s) are listed separately in Tables 3 and 4.

From Figure 11 and Table 3, we can find that the east and north positioning errors are greatly reduced whether outside or in the tunnel by using the IMM-based fusion algorithm. From Table 4, it can be seen that the positioning accuracy of IMM-based fusion algorithm is obviously improved compared with that of the RFID-based preliminary positioning algorithm. For instance, in 6–74 s when the vehicle is driving in the tunnel, the RMS value of the Euclidean distance error of the IMM-based algorithm is decreased to 1.73 m, *i.e.*, about 39% accuracy improvement over the RFID-based algorithm. The reason is that the in-vehicle sensors provide more accurate and richer vehicle state information to correct the preliminary positioning errors. Meanwhile, the preliminary positioning results are used as the position observations to compensate the accumulation errors of the in-vehicle sensors.

The experiments results show that the IMM-STEKF-based fusion algorithm can achieve better performance than the RFID-based preliminary positioning algorithm. Due to the in-vehicle sensors, the velocity and heading angle information can be provided by the fusion algorithm, and the positioning frequency of fusion algorithm is increased to 10 Hz from the value 1 Hz of the RFID-based algorithm.

#### 5.3.2. Comprehensive Test

To further validate and evaluate the performance of the proposed fusion positioning algorithm (abbreviated as IMM), the comprehensive test scenario containing a straight line situation and a curvilinear situation is set, as shown in Figure 5b. The following representative methods are also investigated for comparison.

(1) The proposed preliminary LMS-federated filter-based positioning method only using RFID (abbreviated as RFID).
(2) The dead reckon method using the low-cost Crossbow MEMS-based IMU-440 inertial sensors sampled at 100 Hz, as well as wheel speed sensors sampled at 100 Hz and an electronic compass sampled at 10 Hz. (abbreviated as DR).
(3) The EKF fusion positioning algorithm (abbreviated as EKF). This method uses the single model (CA model) to realize the fusion of the data from RFID-based preliminary positioning and the in-vehicle sensors.
(4) The STEKF fusion positioning algorithm (abbreviated as STEKF). This method uses the single CA model to realize the fusion.

**Figure 12.**
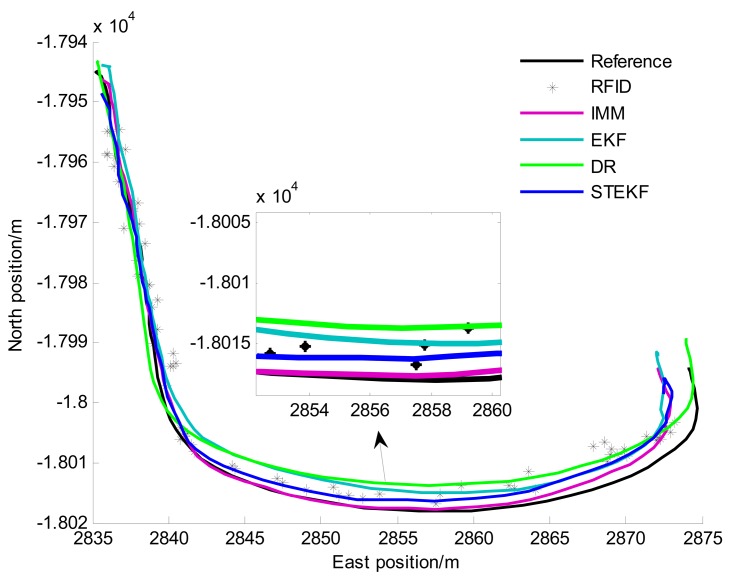
The reference and estimated vehicle trajectories in comprehensive test.

A total of 12 comprehensive tests have been carried out. For brevity, only one test is shown here as an example because similar conclusions can be reached from the other tests. The vehicle trajectories in the comprehensive test are shown in Figure 12.

Table 5 gives the performances of the five positioning methods, RFID, DR, EKF, STEKF and IMM. They include the statistics of Euclidean distance errors (*i.e.*, horizontal position errors), the positioning frequency and the velocity information.

From Table 5, we can find that five methods exhibit different positioning performance. It is clear that the RFID has the worst positioning performance, *i.e.*, both the maximum and RMS values of its Euclidean distance error are the largest. The reason is that the RFID reader can't detect enough tags at the stage when the vehicle is driving outside the tunnel (0–5 s and 70–76 s).

**Figure 13.**
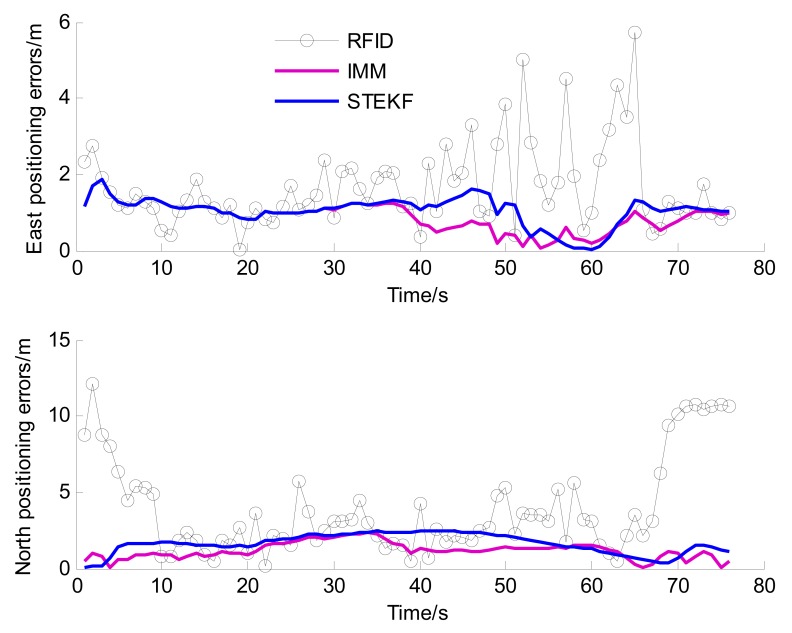
The east and north positioning errors in the comprehensive test.

To verify the positioning performance in the tunnel, we will firstly compare and discuss of RFID, STEKF and IMM. Table 6 shows statistics of the Euclidean distance errors when the vehicle is driving in the tunnel (6–69 s). Figure 13 illustrates the east and north positioning errors of the three methods.

From Table 6 and Figure 13, we can find that the positioning accuracy of STEKF is obviously improved compared with RFID. For example, the RMS value of the Euclidean distance error of STEKF is reduced to 2.19 m from the 3.18 m value of RFID. The reason is that the in-vehicles sensors provide more accurate and richer vehicle state information for fusion positioning, which can remarkably improve the system observability and enhance the positioning reliability. However, since the STEKF only uses a single model, its performance is still poor when the actual driving situation is different from the driving situation of the model.

The IMM can achieve better performance than the STEKF, especially when there exists a curvilinear driving situation. For instance, the RMS value of the Euclidean distance error of IMM is decreased to 1.44 m, *i.e.*, about a 34% accuracy improvement over STEKF. These results verify the effectiveness of the IMM algorithm. This can be attributed to the fact that the CT model of IMM is adapted to the curvilinear driving, and the IMM algorithm can adaptively switch to the CT model when the vehicle is driving in the curve. The STEKF only uses the CA model and is unsuited to the curvilinear driving situation. The errors of STEKF are obviously larger than that of IMM when the vehicle driving in the curve, as shown in Figure 13 (after 35 s).

Therefore, among the three methods the IMM one achieves the optimal accuracy and reliability. Compared with RFID, IMM provides a significant performance improvement, e.g., over 55%. Its positioning frequency is increased to 10 Hz from the value 1 Hz of RFID, and the velocity information can be provided.

As can be seen in Table 5, the STEKF method has the better positioning performance than EKF since there are unrealistic uncertainties in the noise description in the experiments. The DR has worse positioning performance due to the accumulated errors. The proposed IMM method can remarkably improve the system observability by using the RFID-based preliminary positioning results to compensate the accumulated errors.

The low-cost GPS is the most widely used vehicle positioning sensor with accuracies (1σ) of about 3 m for position. From Table 5, we can find that the positioning accuracy of IMM is approximately the same as that of low-cost GPS with higher frequency. The positioning performance of the proposed strategy can meet the requirements of vehicle positioning in tunnels when low-cost GPS is unavailable.

## 6. Conclusions

To realize accurate and reliable positioning for vehicles in tunnels, this paper has presented a multi-sensor fusion strategy, which integrates low-cost sensors, including MEMS-based inertial sensors, wheel speed sensor, electronic compass, and RFID.

In the proposed strategy, both a RFID-based preliminary positioning algorithm and IMM-STEKF-based global fusion algorithm have been developed. First, a LMS-Federated filter is designed to obtain preliminary position information as the observation for the subsequent global fusion. Further, the IMM-STEKF algorithm has been proposed to realize the global fusion. The IMM-STEKF algorithm is designed to fusion multiple observation sources with different sample rates to achieve better performance. Through real-world experiments, the proposed strategy has been evaluated and compared with other representative methods. For the proposed strategy, the effectiveness of both its RFID-based preliminary positioning and global fusion algorithms has been comprehensively verified. During GPS outages in the tunnel, the proposed strategy has shown more obvious advantages and achieved more accurate and reliable performance compared with other methods.

The proposed vehicle positioning strategy in tunnels can be adapted to other GPS-denied environments such as urban areas. It should be noted that, the experiments have only been conducted in an outdoor environment rather than the real enclosed environments due to the limitations of our experimental conditions. In the enclosed environments, the multipath phenomenon may seriously affect the positioning performance. Our future work will be concerned with how to solve this problem, and how to reduce the number of tags for cutting costs with very little sacrifice of accuracy.

## Figures and Tables

**Table 1.** The mean and standard deviation of the RSS fitting errors in different situations.

**Situation**	**Mean**	**STD**
Laboratory	0.8112	8.1897
Outdoor test site	−0.5478	8.2784
Tunnel	1.3741	10.4355

**Table 2.** Statistics of Euclidean distance errors (unit: m).

**Algorithm**	**Max**	**RMS**
LMS-Federated	7.33	3.01
multilateration	11.17	5.72

**Table 3.** The mean and standard deviation of east and north positioning error (unit: m).

**Test Scenario**	**RFID-Based Preliminary Positioning**				**IMM-Based Fusion Positioning**			

	**East Errors**		**North Errors**		**East Errors**		**North Errors**	
			
	**Mean**	**STD**	**Mean**	**STD**	**Mean**	**STD**	**Mean**	**STD**
Whole Test (1–81 s)	1.39	0.86	4.18	4.75	1.31	0.29	1.63	0.56
In tunnel test (6–74 s)	1.21	0.84	2.35	2.32	1.20	0.38	1.50	0.47

**Table 4.** Statistics of the Euclidean distance errors in different tests (unit: m).

**Test Scenario**	**RFID-Based Preliminary Positioning**		**IMM-Based Fusion Positioning**	
	
	**Max**	**RMS**	**Max**	**RMS**
Whole Test (1–81 s)	19.01	4.64	3.90	2.12
In tunnel test (6–74 s)	8.57	2.82	2.87	1.73

**Table 5.** The positioning performance of different methods.

**Method**	**Statistics of Euclidean Distance Errors (unit: m)**		**Velocity Information**	**Positioning Frequency (unit: Hz)**

	**Max**	**RMS**		
RFID	12.43	4.50	No	1
DR	9.48	4.04	Yes	10
EKF	5.77	3.62	Yes	10
STEKF	3.16	2.31	Yes	10
IMM	2.03	1.45	Yes	10

**Table 6.** Statistics of the Euclidean distance errors when the vehicle is driving in the tunnel.

**Method**	**Statistics of Euclidean Distance Errors (unit: m)**	

	**Max**	**RMS**
RFID	6.70	3.18
STEKF	2.98	2.19
IMM	2.07	1.44
